# Photopharmacological Applications for Cherenkov Radiation Generated by Clinically Used Radionuclides

**DOI:** 10.3390/ijms22169010

**Published:** 2021-08-20

**Authors:** Melanie Krebs, Alexander Döbber, Theo Rodat, Ulf Lützen, Yi Zhao, Maaz Zuhayra, Christian Peifer

**Affiliations:** 1 Institute of Pharmacy, Christian-Albrechts-University of Kiel, Gutenbergstraße 76, 24118 Kiel, Germany; mkrebs@pharmazie.uni-kiel.de (M.K.); adoebber@pharmazie.uni-kiel.de (A.D.); trodat@pharmazie.uni-kiel.de (T.R.); 2 Clinic for Nuclear Medicine, Molecular Image Diagnostics and Therapy, Arnold-Heller-Straße, 24105 Kiel, Germany; Ulf.Luetzen@uksh.de (U.L.); Yi.Zhao@uksh.de (Y.Z.); mzuhayra@nuc-med.uni-kiel.de (M.Z.)

**Keywords:** Cherenkov radiation, kinase inhibitor, photopharmacology, CDK2, bio-optical window, radionuclides

## Abstract

Translational photopharmacological applications are limited through irradiation by light showing wavelengths within the bio-optical window. To achieve sufficient tissue penetration, using wavelengths >500 nm is mandatory. Nevertheless, the majority of photopharmacological compounds respond to irradiation with more energetic UV light, which shows only a minor depth of tissue penetration in the µm range. Thus, we became interested in UV light containing Cherenkov radiation (CR) induced as a by-product by clinically employed radionuclides labeling specific tissues. Therefore, CR may be applicable in novel photopharmacological approaches. To provide evidence for the hypothesis, we verified the clinically established radionuclides ^68^Ga and ^90^Y but not ^18^F in clinically used activities to be capable of generating CR in aqueous solutions. We then investigated whether the generated CR was able to photoactivate the caged kinase inhibitor cagedAZD5438 as a photoresponsive model system. Herein, 21% uncaging of the model system cagedAZD5438 occurred by incubation with ^90^Y, along with a non-specific compound decomposition for ^68^Ga and partly for ^90^Y. The findings suggest that the combination of a clinically employed radionuclide with an optimized photoresponsive agent could be beneficial for highly focused photopharmacological therapies.

## 1. Introduction

A recent strategy to overcome specificity issues of pharmacologically active compounds includes photopharmacological approaches, in which the biological activity of a compound can be regulated by the irradiation of light [1]. Using light-responsive molecules, temporal and spatial control and thus high precision in a therapeutic treatment could be achieved [2,3,4]. In line with this notion, Table S1 lists a selection of experimental photopharmacological applications that have been studied recently.

However, the majority of photoresponsive compounds react most effectively to irradiation with light showing wavelengths < 500 nm, typically showing a limiting penetration depth of only micrometers into biological tissue. Moreover, highly energetic UV light can cause harm to cells, resulting, among others, in apoptosis or DNA damage [5,6,7]. On the other hand, the light absorption of hemoglobin and water reaches a minimum between 650 and 900 nm, thus allowing for decent tissue penetration for relevant wavelengths and, as a consequence, for a sufficient translational bioavailability of irradiation. This wavelength range between 650 and 900 nm is commonly referred to as the bio-optical window, and photopharmacological research aims to develop compounds to be activated by irradiation within that range [8,9]. For therapeutically effective applications, the used wavelength of the irradiation should be adjusted ideally within the bio-optical window, and thus, a compound should be photoresponsive within this window, too. However, until today (as shown in Table S1), most photopharmacological approaches use lower wavelengths within the UV range [10,11,12,13,14,15,16,17,18,19,20]. Therefore, an alternative would be highly beneficial to circumvent this limited penetration depth of UV light for successful activation of photoresponsive compounds at deeper tissue levels.

In this context, we became interested in the usage of Cherenkov radiation (CR), which exhibits an emission spectrum in the near-UV range (250–600 nm) and hence covers the wavelength range of most of the photoresponsive compounds published so far (Table S1) [21,22]. Ran et al. already investigated the possibility of activation based on CR generated by ^18^F, and Nakamura et al. reported on CR by ^18^F-FDG for photoimmunotherapy [23]. Furthermore, CR has already been investigated more closely in the field of photodynamic therapy, for example, by Hart et al. for ^90^Y-induced CR [24,25,26,27].

CR can occur as a second form of radiation in addition to ionizing radiation generated from high-energy beta emitters, including clinically established radiopharmaceuticals. These are radiolabeled molecules designed to deliver therapeutic doses of ionizing radiation to specific tumor tissue areas within the body. At specific dose rates, they are used for diagnostics and radiotherapy, thus potentially offering highly interesting combinations for novel photopharmacological applications. CR is commonly known as the emergence of blue light emitted from high-energy radiation sources stored in water in a nuclear power plant. Furthermore, CR is the basic detection principle of a Cherenkov counter, which is a technique for the determination of beta emitting radionuclides used in biochemical assays (e.g., isotope ^32^P) [28,29]. In general, CR can be induced when a charged particle travels faster than light in a dielectric medium (e.g., water). The charged particle locally polarizes the medium molecules, generating excited electronic states. During their return to the ground state, the resulting energy is emitted by the system as CR. This CR typically occurs in an asymmetrical polarization event, the so-called “cone effect”, in which the resulting radiation waves can constructively superimpose each other. If the charged particles in the polarizable medium travel slower than the respective speed of light within the same medium, the resulting radiation waves cancel each other. Therefore, the threshold condition for the induction of CR depends on the refractive index of the polarizable medium as well as on the energy level of the charged particles [30]. The velocity in the respective medium v can be calculated using the following equation, where n is the refractive index of the medium and c is the speed of light in vacuum.

v = c/n(1)

In radiopharmaceutical therapies, especially radionuclides that decay by beta-particle emission are commonly used. Beta decay of the nuclides results in the emission of high-energy electrons or positrons from the nucleus. These locally generated high-energy particles impact the surrounding tumor tissue to consequently kill tumor cells. Accordingly, we focused on the question of whether radiotherapeutical applications generating CR could be useful to activate photoresponsive compounds. Besides beta-emitting radionuclides, external beam radiation commonly used in the clinic for radiation tumor therapy is able to induce CR as well [31], whereby charged particles such as electrons are strongly accelerated in a linear particle accelerator for targeting tumor tissues. The resulting energy is also released in the form of photons, which can induce CR due to Compton scattering. In detail, a photon is scattered by a charged particle such as an electron, which leads to a transfer of energy to the rebounding particle. This energetic recoiling particle is able to induce CR [32,33,34,35].

In the present study, we investigated whether CR, generated by radionuclides or by external irradiation (see SI Section 2.8), could be useful to activate photoresponsive prodrug compounds. Thus, a locally and temporally controlled photopharmacological therapy without the need for external irradiation could be possible. Furthermore, a combination of photoresponsive chemotherapy and radiotherapy could achieve additive effects [10]. Additionally, the amount of necessary radiopharmaceutical activity could be reduced by such a combination, so that both patients and clinical staff would benefit from the concept as the medical applications would possibly be easier to manage. In the present study, from nuclides relevant for diagnostic and therapeutic purposes (Table 1), we took a selection, namely ^18^F, ^68^Ga, and ^90^Y, and aimed to verify whether these radioisotopes were able to produce quantifiable CR in an aqueous medium.

**Figure 1:**
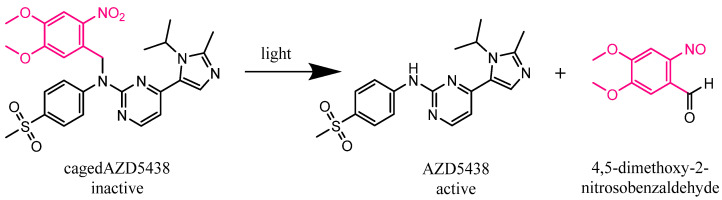
Expected photolysis reaction of the inactive kinase inhibitor AZD5438 caged with the photoremovable protecting group 4,5-dimethoxy-2-nitrobenzyl (DMNB) (cagedAZD5438) to the active AZD5438 and the separated protection group according to Klán et al. [3].

Additionally, we aimed to investigate the potential photocleavage of a caged compound as a photoresponsive model system, both for the selection of the nuclides and for photons and electrons from a clinically used linear particle accelerator (see SI Section 2.8). As the model compound, we chose the highly effective but somewhat unspecific protein kinase inhibitor AZD5438 caged with the well-established photoremovable protecting group 4,5-dimethoxy-2-nitrobenzyl (DMNB) [3,46,47,48] (Figure 1) to obtain a bioinactive prodrug. Protein kinases play an essential role in signal transduction and, in the case of their malfunction, lead to uncontrolled cell growth or reduced apoptosis. This often results in the development of cancer [49,50]. Cyclin-dependent kinases (CDKs) are especially fundamental for a functioning cell cycle and transcription regulation and are investigated as drug targets [51,52,53]. In line with this notion, AZD5438 has been developed as a potent inhibitor of CDKs with the following IC_50_ values: CDK1, 16 nM; CDK2, 6 nM; CDK9, 20 nM [54]. Furthermore, it is an orally bioavailable inhibitor which advanced into clinical studies but failed because of toxic side effects. Hence, the pharmacologically active compound AZD5438 and its inactive caged prodrug cagedAZD5438 may be suitable for highly specific photopharmacological applications, in that the temporal and spatial control of the drug’s activation can greatly decimate the proportion of toxic side effects.

## 2. Results and Discussion

### 2.1. Verification of CR Generated by the Decay of Nuclides in an Aqueous Solution

**Figure 2:**
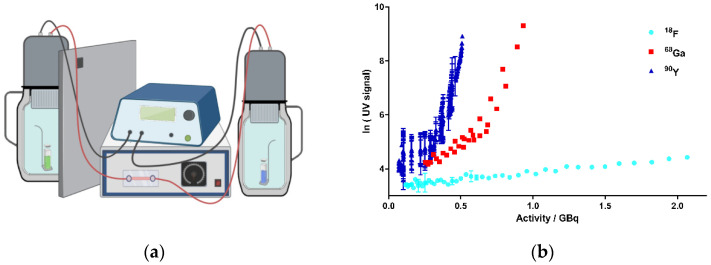
Activity-dependent generation of CR by the decay of ^18^F, ^68^Ga, and ^90^Y in kamiokanne (KK) experiments. (**a**) Schematic test setup of KK experiments. Two thermos flasks separated by a lead wall were filled with water together with a holder for a sealed quartz cuvette containing the samples. The flasks were equipped with photomultiplier tubes (PMTs) and electronic devices to detect CR. The green cuvette represents a solution of the radionuclide sample, and the blue sample illustrates the negative control containing the solvent without radionuclides. On top of the thermos flasks, PMTs were attached to high-voltage supplies (figure created with BioRender.com). (**b**) Results from the KK experiments. The activities of the three radionuclides, ^18^F, ^68^Ga, and ^90^Y, are presented individually for each experiment in correlation to the resulting Cherenkov signal intensity (UV signal). The signal strength corresponds to the number of signals at 10-min intervals. Signals of cosmic radiation were used to determine the baseline (blank value). Each radionuclide was tested in three independent experiments; data represent mean ± SD.

First, it was important to provide evidence for the generation of CR by the decay of the above-mentioned radionuclides in aqueous solutions. Among the typical read-out techniques for biochemical assays is a Cherenkov counter, which exploits exactly the principle of the resulting CR by the decay of radionuclides (e.g., ^32^P) [28]. However, such conventional detection methods could not be established for this study because of the obvious difficulties in handling the radioisotopes, including the long contamination times based on their slow decay. Therefore, when looking for alternative methods for the direct detection of CR, we came across a device called “kamiokanne” (KK). The KK is a simplified system based on the method of the “super-kamiokande” (SKK), which is located in Kamioka, Japan [55]. In fact, the SKK is being used to investigate secondary cosmic radiation. The simplified KK is composed of common thermos flasks with attached photomultiplier tubes (PMTs) and internal high-voltage supplies [56]. The KK is able to measure muons from secondary cosmic radiation, thus generating CR as electromagnetic radiation. The PMTs are able to detect this CR, and consequently, an electrical signal is generated as the read-out. Thus, we decided to test the KK for the detection of CR in our experiments (further details can be found in the SI). The adapted set-up of the KK in our lab is shown schematically in Figure 2a. Here, we investigated whether CR generated by the decay of radionuclides was detectable by the KK. From the multitude of possible radionuclides, the therapeutically and diagnostically used beta-emitting radionuclides ^18^F, ^68^Ga, and ^90^Y were chosen as a representative selection of beta emitters showing clinical significance (Table 1). As the key rationale for the selection of these radionuclides, it was important to focus on meaningful half-life values to allow sufficient repetitions of experiments within a reasonable period. Additionally, the decay energy of the respective radionuclides plays an important role. This energy is not constant, but a continuum spectrum is created in which the maximum emitted energy (E_max_) and the average energy (E_mean_) are key parameters (Figure S8) [57,58,59,60].

A critical energy threshold value of 0.26 MeV was determined by Elrick et al. regarding the generation of CR in an aqueous solution [22]. In order to meet the requirements for potential translational purposes of this concept, we tested the radionuclides in energy ranges corresponding to their respective clinical applications. Thus, we performed the experiments in the activity range for ^68^Ga from 1.163 to 0.256 GBq, for ^18^F from 2.317 to 0.117 GBq, and for ^90^Y from 0.510 to 0.071 GBq. Figure 2b summarizes the results of the experiments. Based on the decay of these nuclides, activity-dependent generation of CR could be determined for all chosen nuclides. A significant increase in CR signals was observed for ^68^Ga and ^90^Y. For ^68^Ga, more activity was required compared to ^90^Y to achieve a comparable CR signal according to their respective decay energies. In contrast, only CR slightly above the blank value (negative control, CR from the secondary cosmic radiation) could be determined for ^18^F, although we used activities up to more than 2 GBq. The results were not unexpected, as the E_mean_ emitted by ^18^F was 0.25 MeV and thus did not reach the threshold value for the formation of CR (Table 1) [22]. Taken together, we could establish a strong correlation between the nuclear decay of the selected nuclides and the resulting CR, suggesting ^68^Ga and ^90^Y to be suitable for photopharmacological applications. ^18^F produced CR as well, but to a much lesser extent, which might not be enough to photoactivate our model system cagedAZD5438.

### 2.2. Activation of a Caged Photoresponsive Compound by Cherenkov Radiation as Model System

**Figure 3:**
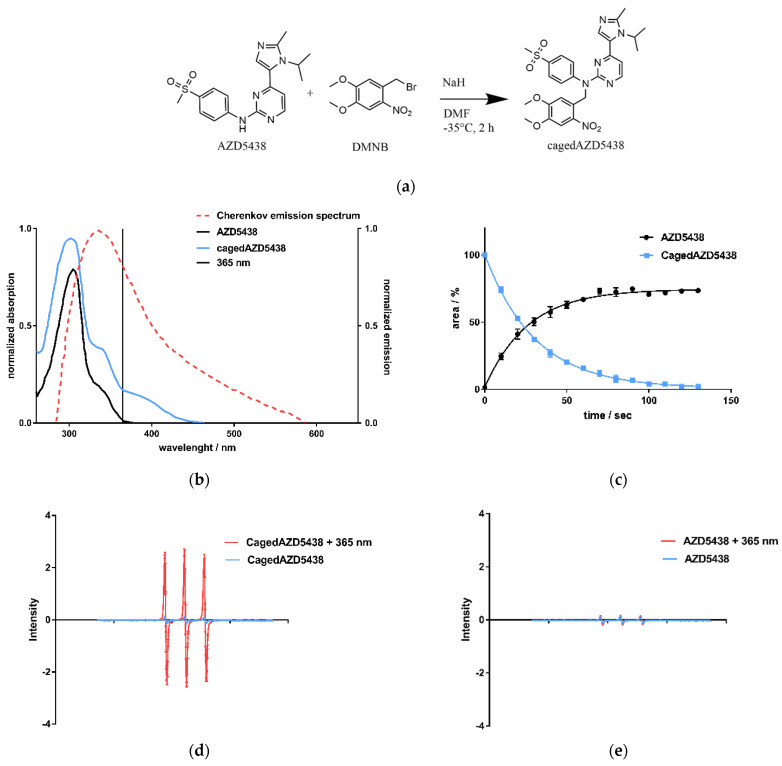
(**a**) CagedAZD5438 was synthesized according to the general procedure for caging of *N*-phenylpyrimidin-2-amines with benzyl bromides [59]. In an S_N_2 reaction, benzyl bromide in dry DMF is added dropwise to AZD5438 at −35 °C under argon atmosphere (yield 55.6%, further details in SI). (**b**) UV/Vis spectra of AZD5438 and cagedAZD5438 with DMNB in aqueous medium with 20% DMSO as solubilizer. An overlay of the Cherenkov emission spectrum (dotted line) illustrates the potential usage for photoactivation, indicating 365 nm as a suitable wavelength for control experiments. Other UV/Vis spectra with different solutions are shown in the SI, Figure S7. (**c**) Photoactivation of cagedAZD5438 by irradiation with UV light at 365 nm; an aqueous compound solution containing 50 μM prodrug cagedAZD5438 (blue line) and 20% DMSO was irradiated at 365 nm, 37.5 mW, every 10 s and then analyzed by HPLC. Repeated experiments with the free inhibitor AZD5438 (Figure S9d) as a control were carried out. Each value is the mean ± SD of four independent experiments. (**d**) Electron spin resonance (ESR) spectra of the spin probe TMTH (1-hydroxy-4-isobutyramido-2,2,6,6-tetramethylpiperidine) incubated with cagedAZD5438 in DMSO without and with irradiation (365 nm, 100 s, 37.5 mW per well). (**e**) ESR spectra of a solution of AZD5438 with and without irradiation (365 nm, 100 s, 37.5 mW per well). As a positive control, the photosensitizer 1,4-naphthoquinone [63] in DMSO was tested under the same conditions. The sample solutions contained the spin probe TMTH (2.5 mM) and 50 μM cagedAZD5438 in 20% DMSO. Error bars indicate SD with *N* = 3.

Having demonstrated the generation of CR by the radionuclides ^18^F, ^68^Ga, and ^90^Y as a side-product, we next investigated whether this process could activate a photoresponsive compound. We selected the 4,5-dimethoxy-2-nitrobenzyl (DMNB) caged AZD5438 (cagedAZD5438) as a novel model compound based on the emission spectrum of the CR and the strong absorption of cagedAZD5438 within the range of 300–400 nm (Figure 3b). The DMNB group was already investigated by our group in connection with the central pharmacophore *N*-phenylpyrimidine-2-amine, a scaffold of many kinase inhibitors, including AZD5438 [61]. Molecular modeling of the X-ray-defined ligand complex of AZD5438 in CDK2 with cyclin E1 (pdb 4FKO) suggested the NH function to be suitable for caging. Herein, due to steric conflicts of the caging moiety, no plausible binding mode for cagedAZD5438 in CDK2 could be determined (see Figure S1) [62].

We achieved the synthesis of cagedAZD5438 by the established general procedure for caging of *N*-phenylpyrimidin-2-amines with benzyl bromides under argon atmosphere and under light protection [59] (Figure 3a). First, we performed preliminary tests to determine the stability of cagedAZD5438 under different conditions (see SI Figures S4 and S5). For the photochemical characterization of cagedAZD5438, we tested the uncaging process upon irradiation. An aqueous solution of 50 µM cagedAZD5438 with 20% DMSO (to avoid solubility issues) was irradiated with light at 365 nm with 37.5 mW, and the samples were subsequently analyzed by HPLC (Figure 3c; for details of the photolysis experiments, see Figure S6). The inhibitor AZD5438 itself proved to be stable during UV irradiation (Figure S9d). In contrast, upon irradiation, cagedAZD5438 was uncaged to produce 75% AZD5438 after 100 s of irradiation. Using electron spin resonance spectroscopy (ESR) investigations, we could confirm that radicals were generated during UV irradiation of a solution of AZD5438. Consequently, a portion of AZD5438 was radically destroyed and thus not detected by the HPLC analysis (Figure 3c–e), in which a mixture of unidentified products occurred.

**Figure 4:**
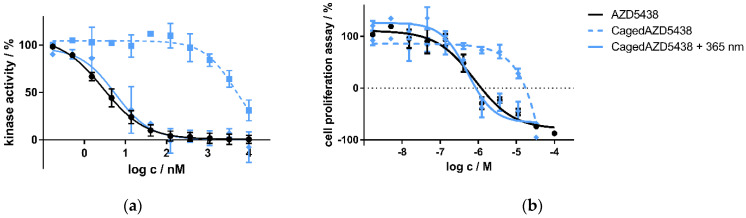
(**a**) Kinase assay of caged and uncaged AZD5438 using the target kinase assay CDK2/cyclin E1 with and without UV irradiation. CDK2 in complex with cyclin E1 was incubated with the inhibitor AZD5438 as well as its caged prodrug cagedAZD5438 to determine kinase activity. In a second setup, 10 min after compound addition both assays were irradiated with UV light for two minutes (365 nm, 37.5 mW). Data represent mean ± SD from two independent experiments, each performed in triplicate. (**b**) Cell proliferation assay of the inhibitor AZD5438 and cagedAZD5438 with and without UV irradiation using Panc89 cells. UV irradiation was performed for 2 min, 365 nm, 37.5 mW. Cell proliferation was determined after 48 h of drug treatment. Data represent mean ± SEM from three independent experiments, each performed in quadruplicate.

Next, we aimed to test the uncaging of cagedAZD5438 in vitro. For this purpose, we initially tested cagedAZD5438 in an enzymatic CDK2/cyclin E1 assay and in a cell proliferation assay under controlled light conditions. In the enzymatic CDK2/cyclin E1 assay, we determined AZD5438 to be highly active with an IC_50_ value of 2.8 nM, which is in the same range as the reference data (IC_50_ = 6 nM) [54]. In contrast, cagedAZD5438 proved to be much less active without irradiation (IC_50_ = 4798 nM). When irradiating the assay containing cagedAZD5438 with 365 nm, the biological activity on CDK2 could be restored, resulting in an IC_50_ value of 5.6 nM (Figure 4a) and achieving a photopharmacological factor of 850 (unirradiated vs. irradiated). Similar results were obtained in the cell proliferation assay using Panc89 cells with resazurin as a read-out. Here, cagedAZD5438 was determined to be biologically inactive with an IC_50_ value >10 μM (Figure 4b). Again, after irradiation with 365 nm, the biological potency of AZD5438 was fully restored, resulting in an IC_50_ value of 0.5 μM (reference AZD5438 IC_50_ = 0.8 μM). Taken together, in both the kinase and the cell assay, we could demonstrate a clear photoresponsive effect for cagedAZD5438, suggesting this compound to be a suitable model system for further experiments involving the radionuclides ^18^F, ^68^Ga, and ^90^Y and for particle accelerator assays (see SI Section 2.8).

### 2.3. ^18^Fluor

**Figure 5:**
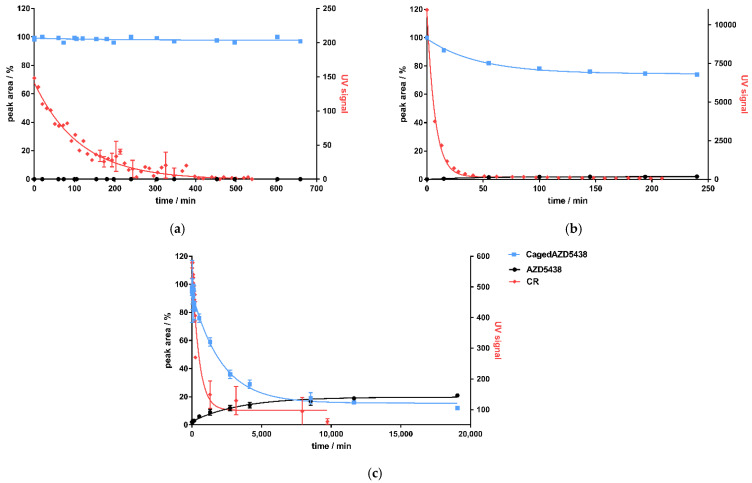
Correlation of CR detected by the KK method as UV signal (red dots) and uncaging of cagedAZD5438 (blue line) by HPLC analysis to yield uncaged AZD5438 (black) for 3 isotopes: (**a**) ^18^Fluor, (**b**) ^68^Gallium, and (**c**) ^90^Yttrium. All curves are fitted with one-phase decay.

As discussed above, and in contrast to the other tested nuclides ^68^Ga and ^90^Y, in our settings, the CR signals generated by the decay of ^18^F were at the baseline level of cosmic ray signals in the KK experiments. However, ^18^F was already reported to be used for activating photoresponsive compounds in a photodynamic therapy approach using sensitizers to be activated by CR produced by ^18^F [24]. Furthermore, in 2012, Ran et al. showed that CR induced by ^18^F mediates uncaging. The authors demonstrated in a proof-of-concept study that a caged derivative of luciferin was uncaged by ^18^Fluorodeoxyglucose (^18^FDG, used for positron emission tomography (PET) in the clinic) in a mouse breast cancer model [23]. Conceptually, the decay of ^18^F showing 96.7% positron emission represents a borderline case with a maximum decay energy of 0.63 MeV and a mean energy of 0.25 MeV, which is very close to the threshold value of 0.26 MeV for the generation of CR [22,36]. However, we incubated cagedAZD5438 with ^18^F and ^18^FDG (20–300 MBq/mL) [12,13,61] for 24 h each, and both the caged and the uncaged species were quantified by HPLC analysis at intervals of 15 min (Table 2). Since cagedAZD5438 has a low solubility in water, we decided to use either DMSO or MeOH as the solvent. In line with the KK results showing no significant generation of CR, under the applied conditions, we observed no uncaging of cagedAZD5438 during incubation with the beta emitter ^18^F for up to 12 h (Figure 5a). In negative controls, the free inhibitor proved to be stable during irradiation (Figure S9a). In order to provide control experiments following the 24-h incubation time, we irradiated the ^18^F samples containing cagedAZD5438 with UV light of 365 nm. Here, strong photoactivation of cagedAZD5438 was demonstrated, suggesting the CR produced by the decay of ^18^F is not sufficient for the uncaging process.

### 2.4. ^68^Gallium

Like ^18^F, ^68^Ga is a beta plus emitter showing 87.7% positron emission. However, the decay of ^68^Ga produced a significantly higher maximum decay energy of 1.9 MeV, and ^68^Ga had an E_mean_ level of 0.84 MeV, considerably above the threshold of 0.26 MeV to generate CR. Thus, we incubated freshly prepared ^68^Ga samples ranging from 300 to 900 MBq with a solution of 50 µM cagedAZD5438 to investigate its photoactivation by HPLC (Table 3). Due to its short half-life, ^68^Ga was generated in situ in the lab and could be eluted directly from its parent isotope ^68^Ger using 1M HCl (^68^Ga generator).

Correlating to the detection of CR produced by ^68^Ga, the amount of cagedAZD5438 in the samples decreased to approximately 75%, reaching a plateau at 149 min (Figure 5b). A non-quantitative uncaging was already observed by the photochemical characterization of cagedAZD5438, yielding only ca. 75% AZD5438 (Figure 3c). However, during incubation of the samples with ^68^Ga, the concentration of AZD5438 did not increase correspondingly. In fact, the HPLC analysis showed a rather complex product mixture, suggesting an alternative degradation reaction by strong CR irradiation, by the emitted positrons, or by radical processes. Assays with a solution of AZD5438 incubated with ^68^Ga did not indicate any destruction (Figure S9b). In contrast, we detected a partial destruction of cagedAZD5438 in the UV experiments (Figure 3c), which, according to the ESR experiments (Figure 3d,e), belongs to radical processes. However, direct ESR experiments employing ^68^Ga were not possible due to the specific handling requirements of the radioactive samples. For the ^68^Ga experiments, although we could demonstrate production of CR, we observed no release of AZD5438 from cagedAZD5438. Instead, a 25% decrease in cagedAZD5438 was detected, reaching a plateau at 149.3 min for CR (produced by the ^68^Ga decay) and at 148.8 min for the decomposition of cagedAZD5438, showing a strong correlation (Figure 5b). Thus, these findings suggest an uncaging reaction mediated by the CR but also compound decomposition by positrons generated by the decay of ^68^Ga.

### 2.5. ^90^Yttrium

In contrast to ^18^F and ^68^Ga as positron emitters being used in radiopharmaceutical diagnostics, ^90^Y is involved in radiotherapy (Table 1). The radionuclide ^90^Y is a pure β-emitter possessing a decay energy of E_mean_ = 2.28 MeV and a half-life time of 64 h. We aimed to investigate whether uncaging of cagedAZD5438 can be achieved by the decay of ^90^Y. Therefore, we incubated a 50 μM solution of cagedAZD5438 with a solution of ^90^Y showing 419 MBq containing 20% DMSO in a total volume of 1.25 mL (Table 4). As a negative control, we used the uncaged AZD5438 accordingly (Figure S9c), and as positive controls, samples of cagedAZD5438 were irradiated with UV light of 365 nm. The samples of cagedAZD5438 incubated with ^90^Y showed an uncaging reaction producing 21% AZD5438, reaching a plateau after 7 d (Figure 5c).

In contrast to the experiments with ^68^Ga and ^18^F, a release of 21% AZD5438 resulted by incubating cagedAZD5438 with the β-emitter ^90^Y over a period of approx. 14 days (Figure 5c). However, besides the CR-mediated uncaging of cagedAZD5438 and similar to the ^68^Ga experiments (Figure 5b), a significant compound decomposition also occurred, which may be dependent on the β-decay of ^90^Y.

## 3. Conclusions

In this study, we verified experimentally using the KK method that the decay of the clinically applied beta emitters ^68^Ga and ^90^Y generates CR in an aqueous solution. A direct comparison of the main decay energies of the investigated nuclides showed that ^18^F with 0.25 MeV releases significantly less energy than ^68^Ga with 0.84 MeV and ^90^Y with 0.93 MeV. We next employed the novel photoresponsive CDK2 inhibitor prodrug cagedAZD5438 as a model system to investigate whether the CR was able to uncage the prodrug when incubated together with the radionuclides. In correlation to the minor CR produced by the β+ emitter ^18^F, no uncaging could be determined for cagedAZD5438. Incubation of cagedAZD5438 with the β+ emitter ^68^Ga partly resulted in a complex product mixture. Furthermore, incubation of cagedAZD5438 with the β-emitter ^90^Y yielded 21% AZD5438 but also indicated a compound decomposition towards a complex mixture. These findings suggest photoactivation of the photoresponsive model compound cagedAZD5438 by CR but also compound decomposition by the β+/β− irradiation produced by the decay of ^68^Ga and ^90^Y, respectively. Based on this proof-of-concept study, an optimized design of photoresponsive compounds could use CR for prodrug activation while providing stability against the β+/β− irradiation. Thus, CR induced by therapeutic radiopharmaceuticals such as ^90^Y could offer a possibility for a synergistic combination of radiotherapy with targeted photopharmacology in future applications.

## Figures and Tables

**Table 1:** Parameters of selected radionuclides with clinical significance [36]. Every positron-emitting (ß+) nuclide results in electron capture (ec). This is another mode of beta decay in which an electron is captured by the atomic nucleus [37]. Iβ(abs) *: absolute β^−^ or β^+^ intensity in %.

Nuclide	$t\frac{1}{2}$	E_max_/MeV	E_mean_/MeV Iβ(abs) *	Type of Radiation (Branching Ratio)	Indication
^68^Ga	67.71 min	1.9	0.84 (87.72%)	ec ß+ (100%)	Diagnostics, PET/CT [38]
^18^F	109.77 min	0.63	0.25 (96.73%)	ec ß+ (100%)	Diagnostics, PET/CT [39]
^153^Sm	46.28 h	0.81	0.23 (49.40%)	ß− (100%)	Bone metastases [40]
^90^Y	64.00 h	2.28	0.93 (99.99%)	ß− (100%)	Neuroendocrine tumors, pleural and peritoneal carcinosis, hepatocellular and cholangiocellular carcinoma, liver metastases, rheumatoid arthritis and activated arthrosis, malignant lymphomas of B cells [40,41]
^186^Re	3.72 d	1.07	0.36 (70.99%)	ß− (92.33%)	Bone metastases, rheumatoid arthritis, activated arthrosis [41]
^177^Lu	6.64 d	0.50	0.15 (79.44%)	ß− (100%)	Neuroendocrine tumors [42], bone pain palliation [43], metastatic castration-resistant prostate cancer [44]
^131^I	8.03 d	0.61	0.19 (89.60%)	ß− (100%)	Neuroendocrine tumors, pleural and peritoneal carcinosis [40], thyroid autonomy, Graves’ disease, goiter, thyroid cancer, pheochromocytoma, liver metastases, liver cell and cholangiocellular carcinoma [41]
^169^Er	9.39 d	0.35	0.10 (55.50%)	ß− (100%)	Rheumatoid arthritis [45]
^32^P	14.27 d	1.71	0.70 (100.00%)	ß− (100%)	Polycythemia vera [40,41], essential thrombocythemia [41]

**Table 2:** Trials from cagedAZD5438 activation experiments using ^18^F and ^18^FDG as the radioactive source. As a solubilizer, either DMSO or MeOH was added. Each sample was analyzed by HPLC every 15 min for up to 24 h in two independent experiments. Following incubation with ^18^F and ^18^FDG showing no uncaging of cagedAZD5438, samples 1–5 were irradiated with UV light, yielding AZD5438 (positive controls).

Sample	Radioactivity/MBq	Solvent	Total Volume/mL	Compound Concentration/μM
**1**	20	10% DMSO in water	1	50
**2**	200	10% DMSO in water	1	50
**3**	150	10% DMSO in water	1	50
**4**	107	50% MeOH in water	0.4	125
**5**	120	50% MeOH in water	0.4	125

**Table 3:** Incubation of cagedAZD5438 with the beta plus emitter ^68^Ga under varying conditions (samples 1–6; in samples 3 and 4, the acidic pH was buffered with ammonium acetate and HEPES). Each sample was analyzed twice. As positive controls, after incubation with ^68^Ga, samples 1–6 were irradiated with UV light, showing uncaging of cagedAZD5438. As a negative control, a solution of the non-radioactive ^69^Ga was employed (sample 6). Incubating a solution of AZD5438 under the respective conditions used for samples 1–6 proved the inhibitor AZD5438 to be stable (Figure S9b).

Sample	Radioactivity/MBq	Solvent	Total Volume/mL	Compound Concentration/μM
**1**	300	20% DMSO in 1 M HCl	1.25	50
**2**	900	20% DMSO in 1 M HCl	1.25	50
**3**	900	20% DMSO in 1 M HCl with 0.4 M NH_4_Ac	1.25	50
**4**	900	20% DMSO in 1 M HCl with 5 M HEPES	1.25	50
**5**	900	20% DMSO in 1 M HCl	3	50
**6**	–	20% DMSO in 1 M HCl and ^69^Ga	1.25	50

**Table 4:** Activation of cagedAZD5438 upon incubation with the beta minus emitter ^90^Y (*N* = 3). As positive controls, samples of cagedAZD5438 were irradiated with UV light, proving quantitative uncaging.

Sample	Radioactivity/MBq	Solvent	Total Volume/mL	Compound Concentration/μM
**1**	419	20% DMSO in 0.04 M HCl	1.25	50
**2**	–	20% DMSO in 0.04 M HCl	1.25	50

## Data Availability

Not applicable.

## Supplementary Materials

The following are available online at https://www.mdpi.com/article/10.3390/ijms22169010/s1.
