# Supplementary material for: Photopharmacological Applications for Cherenkov Radiation Generated by Clinically Used Radionuclides
Source: Int J Mol Sci. 2021 Aug 20;22(16):9010. doi: 10.3390/ijms22169010 (PMC8396513; doi:10.3390/ijms22169010)
Supplement: Supplementary file 1 [file ijms-22-09010-s001.zip › ijms-1266917-supplementary.pdf]

Supporting Information

Photopharmacological Applications for Cherenkov Radiation Generated by Clinically Used Radionuclides

Melanie Krebs [1], Alexander Döbber [1], Theo Rodat [1], Ulf Lützen [2], Yi Zhao[2] Maaz Zuhayra [2], Christian Peifer [1]

[1] Pharmaceutical Institute of Kiel, Gutenbergstraße 76, 24118 Kiel, Germany
[2] Clinic for Nuclear Medicine, Molecular Image Diagnostics and Therapy, Arnold-Heller-Straße, 24105 Kiel, Germany

Table of Contents

1 Published photopharmacological approaches using UV light 2

2 Experimental section 3

2.1 Molecular modeling 3

2.2 Reagents 4

2.2.1 Nuclides 4

2.2.2 AZD5438 4

2.3 Synthesis of **cagedAZD5438** 5

2.3.1 NMR 7

2.3.2 HPLC analytics 7

2.3.3 MS 7

2.4 Photochemical Characterization 8

2.4.1 Stability tests 8

2.4.2 Photolysis Experiments 9

2.4.3 UV/Vis spectroscopy 10

2.5 Biological Evaluation 11

2.5.1 Kinase assay 11

2.5.2 Cell proliferation assay 11

2.6 Kamiokanne 12

2.7 Radioactive experiments 13

2.7.1 HPLC analysis 13

2.7.2 Beta Spectra of the chosen nuclides ^{18}F, ^{68}Ga and ^{90}Y 13

..... 13

2.7.3 ^{18}F experiments 14

2.7.4 ^{18}FDG experiments 14

2.7.5 ^{68}Ga experiments 14

2.7.6 ^{90}Y experiments 14

2.7.7 Stability tests of AZD5438 with the nuclides and under irradiation with 365 nm 15

2.8 Linear particle accelerator experiments 16

2.8.1 Implementation 17

2.9 ESR experiments 18

1 Published photopharmacological approaches using UV light

Supplemental Table S1. Examples of published photopharmacological concepts involving photoresponsive small molecules. The photoactivation by light of a determined wavelength typically takes place either by irreversible cleavage of a photolabile protective group ("caged" photoactivatable prodrugs) or by reversible photoinduced E/Z isomerization of a photoswitchable compound.

Sample	Compound	Target	Type of Photoactivation	Wavelength	Reference
1	1,2 dithienylethene (DTE) inhibitor	hCAI	Photochemical ring changing, in vitro	312/ > 434 nm	Vomasta et al., 2008
2	Caged Rho kinase inhibitor	Rho kinase	Photoactivatable prodrug, in vivo	365 nm	Morckel et al., 2012
3	Caged imatinib	BCR-ABL kinase	Photoactivatable prodrug, in vitro	365 nm	Zindler et al., 2015
4	Caged RET inhibitors	Tyrosine-protein kinase receptor RET	Photoactivatable prodrug, in vivo	365 nm	Bliman et al., 2015
5	RET inhibitor derivative	RET	Photoswitchable, in vitro	365 nm	Ferreira et al., 2015
6	Second messenger lipid diacylglycerol (DAG)	PKC	Photoswitchable, in vivo	360 nm	Frank et al., 2016
7	PKC inhibitor derivative	PKC	Photoactivatable prodrug, in vitro	490 nm	Wilson et al., 2017
8	Axitinib	VEGFR2	Photoswitchable, in vitro	365/385 nm	Schmidt et al., 2018
9	Caged doxycycline analogues		Photoactivatable prodrug	405 nm	Goegan et al., 2018
10	Vemurafenib	BRAFV 600E	Photoactivatable prodrug, in vitro	365 nm	Hoorens et al., 2019
11	Lidocaine derivative	Potassium channel	Photoswitchable, PCC	400/530 nm	Trads et al., 2019
12	AZD5438	CDK1/2/9	Photoactivatable prodrug, in vitro	365 nm	Present work

2 Experimental section

2.1 Molecular modeling

Molecular docking studies were performed on a Dell (1 Dell Way, Round Rock, TX 78664, USA) system including an Intel Xeon E5620 at 2.40 GHz, 24 GB RAM, and a 64-bit Windows 7 Enterprise. Schrödinger Maestro Version 11.0, LLC, New York, USA, was used as the molecular modeling software. First, the CDK2 cyclin E1 structure (pdb: 4FKO1) was imported and processed with Protein Preparation Wizard. Missing side-chains or loops were added using Prime version 4.6. The structure was pre-processed, and waters with less than three H-bonds to non-waters were removed and optimized using PROPKA at a pH of 7.0. Alternatively, MacroModel version 11.4015 was used for ligand preparation. Finally, the protein structure was minimized with the OPLS3 force field. Ligands were processed with LigPrep version 4.0015. For ligand minimization, we chose the OPLS3 force field, with Epik version 3.8015 generating states at a pH of 7.0 ± 2.0 and at most 32 states per ligand. For receptor grid generation, the ligand was picked and Van der Waals radius scaling was set to a factor of 1.0 with a partial charge cut-off of 0.25. Grid generation and minimized ligands were integrated in the Glide ligand docking module which was run in extra precision mode. For visualization, the superposition tool was used to align the structure of cagedAZD5438.

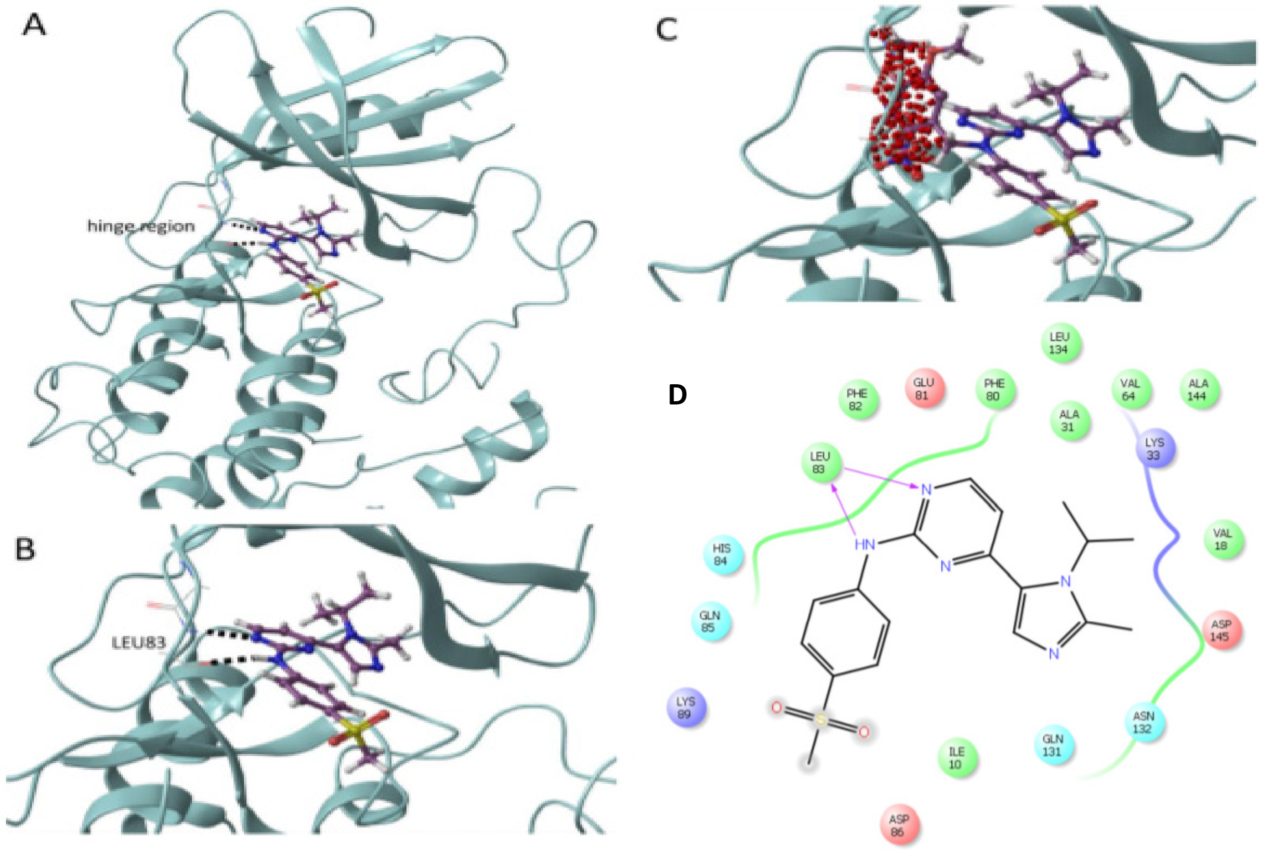

Supplemental Figure S1. Three-dimensional binding mode of inhibitor AZD5438 in the ATP binding pocket of CDK2 predicted by molecular modeling studies (pdb 4FKO). (**A**) H-bonds formed between the ligand and the protein backbone are indicated by black dotted lines. (B) Enlarged view of the binding mode. (**C**) Superimposed DMNB cagedAZD5438 in the ATP binding pocket. Red dashed lines represent sterical clashes between the PPG and the CDK2. (**D**) Two-dimensional ligand interaction diagram of AZD5438. H-bond interactions of the ligand towards the protein backbone are shown in purple. Solvent exposure is highlighted in grey.

2.2 Reagents

2.2.1 Nuclides

^{90}Y was obtained from the commercial company f-con Eckert & Ziegler (Strahlen- und Medizintechnik AG, Berlin, Germany) and ^{18}F from Life Radiopharma f-Con GmbH (Holzhausen a. d. Haide, Germany). They were used without further purification. ^{68}Ga was generated in situ at the UKSH Kiel in the department of nuclear medicine and radiopharmacy using a ^{68}Gallium generator from the company Eckert & Ziegler (Strahlen- und Medizintechnik AG, Berlin, Germany).

2.2.2 AZD5438

The inhibitor AZD5438 was purchased from MedChemExpress USA (MedChemExpress LLC, 1 Deer Park Dr, Suite Q, Monmouth Junction, NJ 08852, USA). Before usage, the compound was purified and fully characterized. Purification was achieved using RP flash chromatography with a gradient of water and methanol to yield a white powder (100% HPLC purity).

2.3 Synthesis of cagedAZD5438

***N*-(4,5-dimethoxy-2-nitrobenzyl)-4-(1-isopropyl-2-methyl-1*H*-imidazol-5-yl)-*N*-(4-(methylsulfonyl)phenyl)-pyrimidin-2-amine**

CagedAZD5438 was synthesized according to the general procedure for caging of the *N*-phenylpyrimidin-2-amine moiety in AZD5438 (100 mg, 0.27 mmol) with 4,5-dimethoxy-2-nitrobenzylbromide (82 mg, 0.30 mmol). Sodium hydride (1.2 equiv.) was dissolved in 5 mL of dry DMF under an argon atmosphere. After cooling to 0°C, the *N*-phenylpyrimidin-2-amine AZD5438 (1.0 equiv.), dissolved in 3 mL of dry DMF, was added dropwise to the reaction. After 1 h, the reaction was cooled to -35°C, and 4,5-dimethoxy-2-nitrobenzylbromide (1.1 equiv.), dissolved in 3 mL of dry DMF, was added to the reaction dropwise. After stirring at -35°C for 1 h, the reaction was quenched by slow addition of H_2O. After 1 h, the reaction was filtrated. The precipitate was further purified by RP flash chromatography using a gradient of methanol and water to afford the particular test compound cagedAZD5438 *N*-(4,5-dimethoxy-2-nitrobenzyl)-4-(1-isopropyl-2-methyl-1*H*-imidazol-5-yl)-*N*-(4-(methylsulfonyl)phenyl)-pyrimidin-2-amine as a bright yellow solid (100% HPLC purity).

Yield: 84 mg (0.15 mmol, 55.6%)

^{1}H NMR: (300 MHz, DMSO-d_6): δ = 1.05 (d, 3JHH = 7.1 Hz, 6H), 2.39 (s, 3H), 3.21 (s, 3H), 3.66 (s, 3H), 3.85 (s, 3H), 5.32 (pent, 3JHH = 7.0 Hz, 1H), 5.65 (s, 2H), 6.93 (s, 1H), 7.20 (d, 3JHH = 5.3 Hz, 1H), 7.52 (s, 1H), 7.73 (m, 3H), 7.91 (d, 3JHH = 8.8 Hz, 2H), 8.38 (d, 3JHH = 5.3 Hz, 1H) ppm.

^{13}C NMR: (75 MHz, DMSO-d_6): d = 16.1, 20.6, 43.5, 46.9, 51.4, 56.0, 56.1, 108.6, 109.8, 109.8, 126.6, 127.9, 128.6, 128.8, 133.0, 136.9, 139.8, 147.4, 148.4, 148.8, 153.3, 157.8, 157.9, 160.7 ppm.

HRMS (ESI): m/z = 567.20204 $[M^+H]^+$ (calc. m/z = 567.20203)

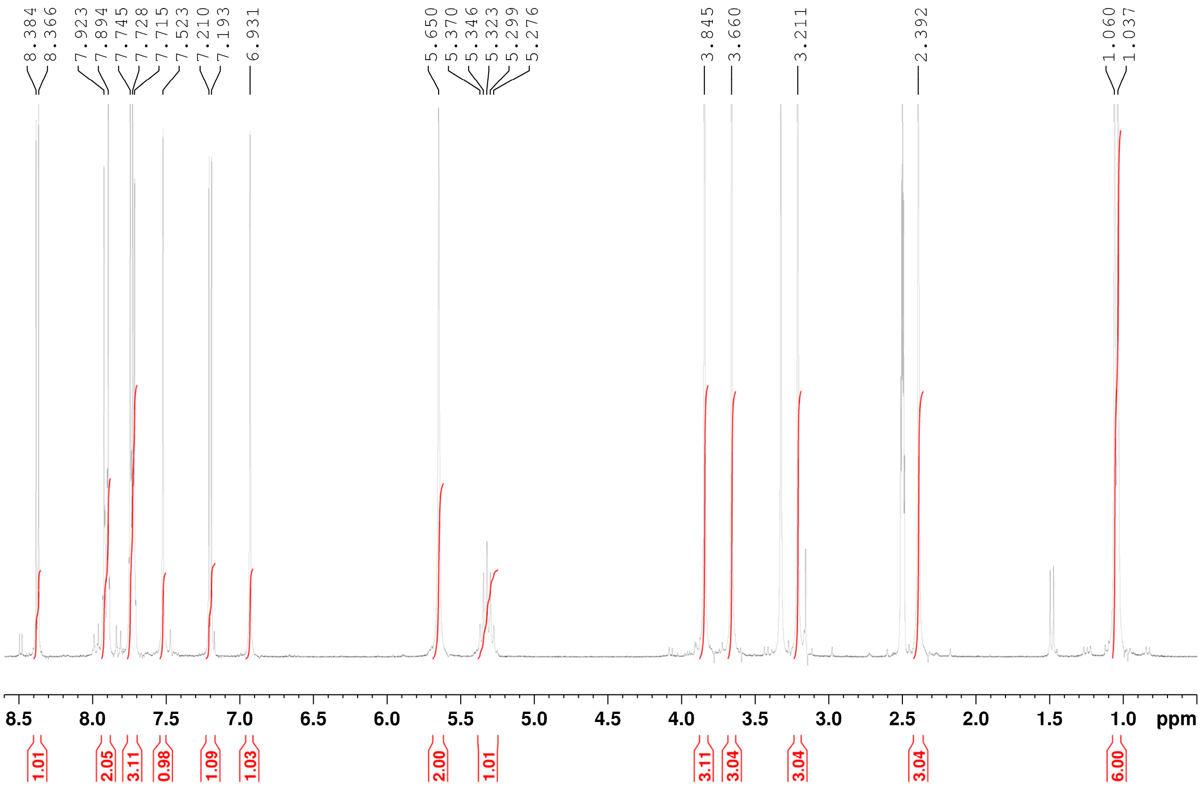

Supplemental Figure S2: ^{1}H-NMR spectrum of cagedAZD5438 in DMSO-d_6.

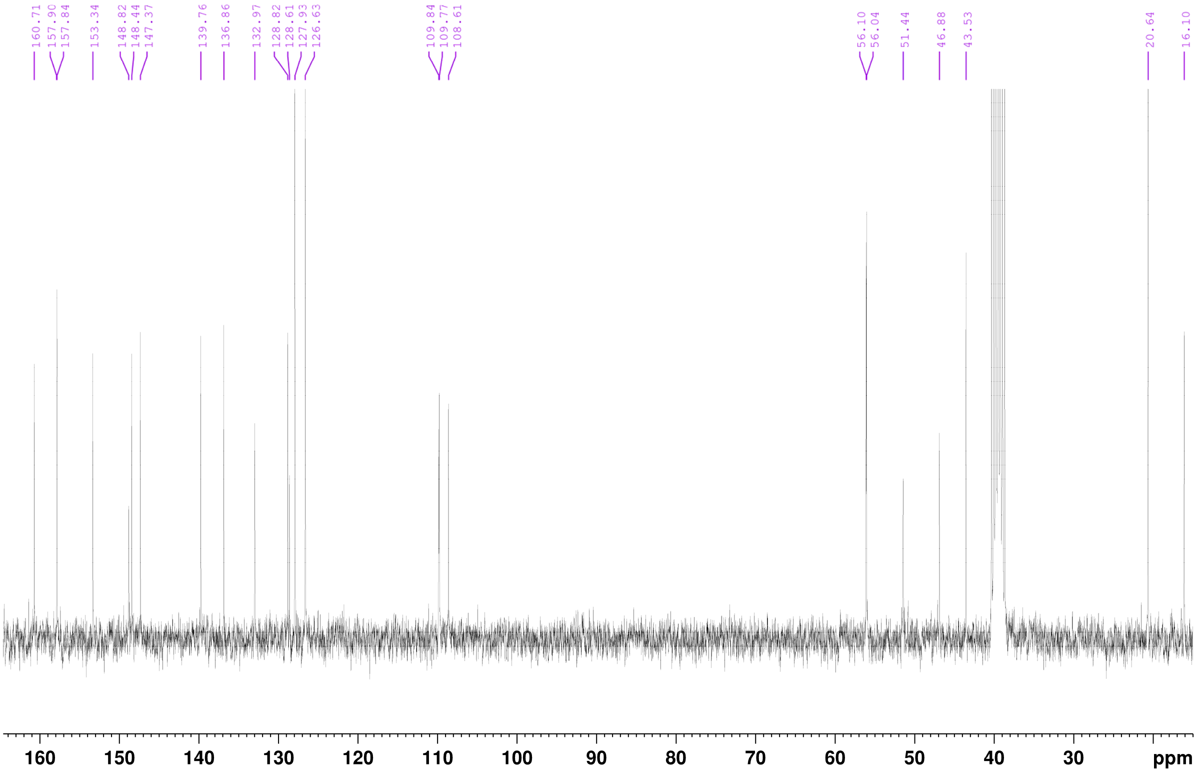

Supplemental Figure S3: ^{13}C-NMR spectrum of cagedAZD5438 in DMSO-d_6.

2.3.1 NMR

NMR spectra were plotted on a Bruker (Bruker Daltonics GmbH & CoKG, Bremen, Germany) Avance III 300 instrument, tempered at 298 K. ^{1}H (300 MHz) and ^{13}C (75 MHz) spectra were referenced to the respective deuterated solvent signals of DMSO-d_6 (δ ^{1}H NMR: 2.50 ppm; δ ^{13}C NMR: 39.52 ppm) as an internal standard. The following shortcuts were used to classify the appropriate signals: s (singlet), d (doublet), pent (pentet), and m (multiplet).

2.3.2 HPLC analytics

HPLC quantification was performed with either an Agilent 1100 series instrument or a Hewlett Packard 1050 Series instrument (for both Agilent and Hewlett Packard: Agilent Technologies, Waldbronn, Germany). The Agilent 1100 series instrument was used with a Phenomenex KinetexQ C8 5-millimeter (150 mm · 4.6 mm) column at 25°C. For elution, different gradients of KH_2PO_4 buffer (0.01 M, pH 2.3)/acetonitrile (5% → 90% MeCN, 1.5 ml/min, 14 min) were used. The detection wavelength was set to 296 nm. For quantification with the Hewlett Packard 1050 Series HPLC, we used either a Kinetex® C8 5-micrometer (150 mm · 4.6 mm, Phenomenex, Aschaffenburg, Germany) or a STAGROMA® C18 5-micrometer column (125 mm · 4 mm, Stagroma AG, Reinach, Switzerland). The mobile phase and detection wavelength were equivalent to quantification with Agilent 1100.

2.3.3 MS

Mass spectrometry was performed on a Bruker Esquire LC ion trap mass spectrometer (Bruker Daltonics GmbH & CoKG, Bremen, Germany) with electron spray ionization (dry gas, 9 L min-1; nebulizer, 35 psi; drying temperature, 350 °C; positive and negative mode). Chromatic separation was achieved with an Agilent 1100 HPLC system (Waldbronn, Germany) with an RP-8 column (Agilent Eclipse XDB-C8, 150 mm length 4.6 mm diameter) and a gradient of 0.1% acetic acid/acetonitrile. The eluent flow rate was 1 ml min^{-1}. High-resolution mass spectrometry was performed either on a Thermo Fisher Q Exative Plus mass spectrometer (Hybrid Quadrupol Orbitrap, Thermo Fisher Scientific, Bremen, Germany) with electron ionization in positive ion mode, a JEOL AccuTOF 4G mass spectrometer with electron impact ionization, or a BIFEXC III mass spectrometer (Bruker Daltonics GmbH & CoKG, Bremen, Germany) including matrix-assisted laser desorption/ionization (4-chloro-α-cyanocinnamic acid matrix, 337 nm ionization, 19 kV acceleration) and a time-of-flight detector.

2.4 Photochemical Characterization

2.4.1 *Stability tests*

Stability tests were carried out under different light conditions, temperatures, concentrations, and solvents.

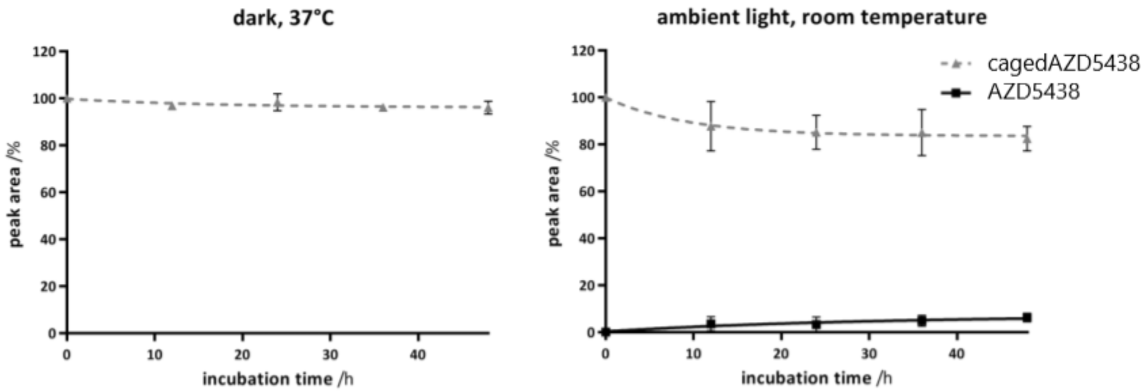

Supplemental Figure S4. Stability of cagedAZD5438 in solution. A compound solution of 100 µM cagedAZD5438 in PBS buffer with 20% DMSO was incubated either in a cell culture incubator in the dark at 37°C (left) or in the lab exposed to ambient light at room temperature (right). After the indicated time points, peak area determination was performed using HPLC analysis. Contents of the caged species are plotted against the uncaged one. Each value is the mean ± SD of two independent experiments.

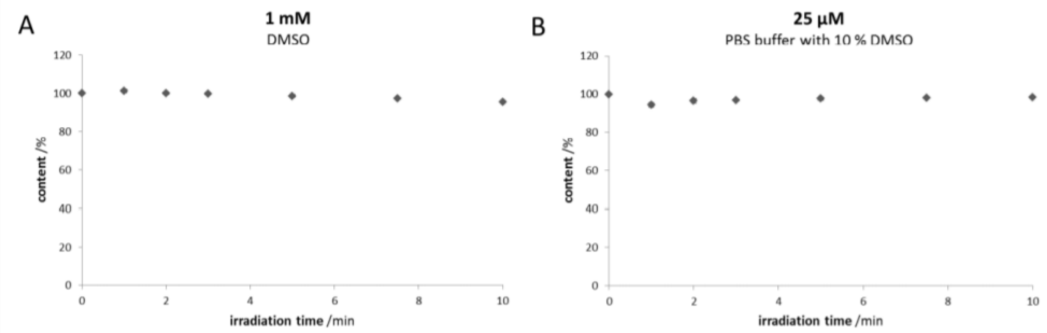

Supplemental Figure S5. UV stability of the inhibitor AZD5438. (**A**) A 1 mM compound solution in DMSO was irradiated at 365 nm (360 mW) in a 96-well plate for up to 10 min. (**B**) A 25 µM compound solution in PBS buffer with 10% DMSO was irradiated at 365 nm (75 mW) in a 96-well plate for up to 10 min. Subsequently, samples of both A and B were analyzed by HPLC within two independent experiments.

2.4.2 *Photolysis Experiments*

The free inhibitor AZD5438 and the caged compound cagedAZD5438 were examined as an aqueous solution (50 µM) with 20% DMSO. Briefly, 100 µL of each solution was irradiated in a well of a 96-well plate at 365 nm (LED source: 16x Nichia NCSU276A U365, Sahlmann Photochemical Solutions, Bad Segeberg, Germany). The light intensity in each UV experiment with 365 nm was 37.5 mW/well. After 0, 10, 20, 30, 40, 50, 60, 70, 80, 90, and 100 seconds, samples were taken and analyzed by HPLC at a wavelength of 296 nm without further dilution. Each sample was injected into the HPLC twice.

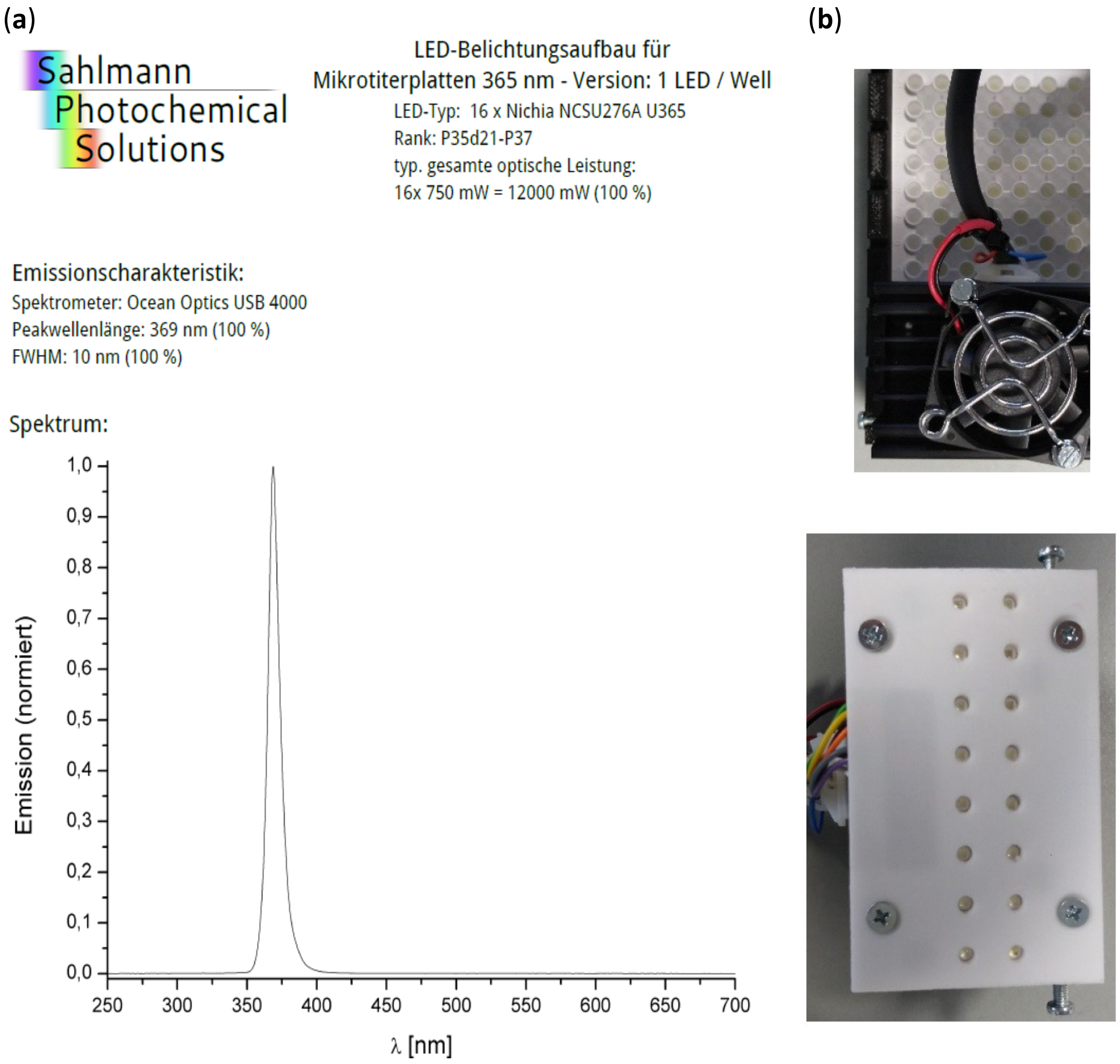

Supplemental Figure S6. (**a**) Technical data of 365 nm light source manufactured and purchased at the company "SahlmannPhotochemicalSolutions" (Bad Segeberg, Germany). LED type 16 × Nichia NCSU276A U365 Rank: P35d21-P37; 16 × 750mW = 12.000 mW (100%); emission spectrum by OceanOptics USB 4000; peak wavelength, 369 nm (100%); FWHM width, 10 nm (100%). (**b**) 365 nm 16 LED lamps (1 LED per well, fitting to a standard 96-well assay plate).

2.4.3 UV/Vis spectroscopy

UV/Vis spectra were acquired with a FLUOstar Omega reader, BMG Labtech (Ortenberg, Germany), in a transparent 96-well plate. The final compound concentration was 0.1 mM.

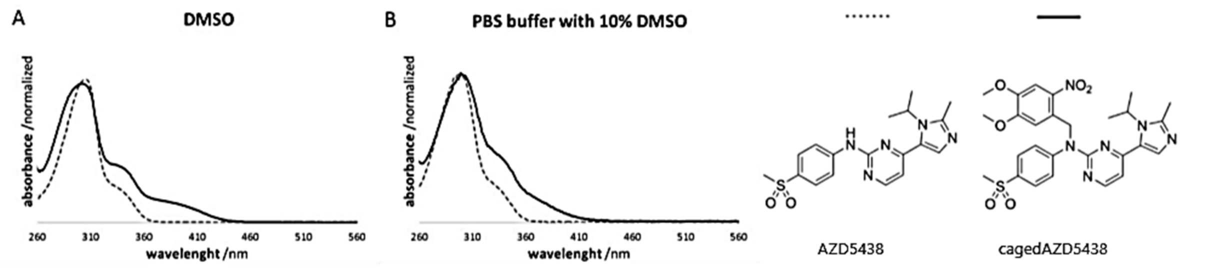

Supplemental Figure S7. UV/Vis absorption spectra. UV spectra of AZD5438 and its caged prodrug were recorded in (**A**) DMSO and (**B**) PBS buffer with 10% DMSO.

2.5 Biological Evaluation

2.5.1 Kinase assay

CDK2 cyclin E1 assays were performed with the ADP-Glo™ Assay kit (Promega GmbH, Walldorf, Germany) according to the manufacturer's manual. The caged inhibitor cagedAZD5438 was dissolved in 10% DMSO and tested in a range of 1×10^{-5} to 2×10^{-10} M, in duplicate. The protein kinase, the respective compound, ATP, and substrate were incubated for 1 h at 30 °C under ambient light exclusion. For the photoactivation experiments, the test plates were irradiated at 365 nm for 3 min (LED source: 16× Nichia NCSU276A U365, 5%, 37.5 mW/well, Sahlmann Photochemical Solutions, Bad Segeberg, Germany). The final concentrations of reagents were 10 µM ATP, 0,1 µg/µl Histone H1 (substrate), and 1% DMSO. Plate read-out was performed with the FLUOstar Omega reader (BMG Labtech). Dose–response curves and IC_{50} values were calculated with GraphPad Prism v.7.03 (GraphPad Software Inc, San Diego, CA, USA) using the Sigmoidal fitting (log(inhibitor) vs. response – variable slope).

2.5.2 Cell proliferation assay

Since CDK2 plays a role in oncogenesis, the candidates were tested on the pancreatic cancer cell line Panc89. Panc89 cells, also known as T3M4, were obtained from Christian Röder, Institute for Experimental Cancer Research, CAU Kiel, Germany. Cells were cultivated at 37 °C, 5% CO_2, in a humidified atmosphere. The Panc89 cells were nourished with Dulbecco's MEM (Lifetech, Life Technologies, Darmstadt, Germany) concentrated with 10% fetal calf serum (Life Technologies, Darmstadt, Germany) and 1 mM pyruvate (Life Technologies, Darmstadt, Germany). For the cell proliferation assay, cells were counted with Cell Scepter (Merck Millipore GmbH, Darmstadt, Germany) and seeded to a final number of 5000–15,000 cells/mL in white 96-well plates (PerkinElmer Cellular Technologies Germany GmbH, Hamburg, Germany). After 24 hours of incubation under the cultivation parameters, compounds were added and irradiated with a 365 nm LED for 5 min, 37.5 mW/well. For dose–response curves, compounds were dissolved in DMSO to a final concentration of 10 mM in assay and diluted in 1:3 steps. A day-zero plate was incubated with resazurin to determine cell proliferation at compound treatment. After additional 48-hour incubation and medium removal, cells were incubated with 2 mg/mL resazurin for two hours. Mitochondria in eukaryotes reduce resazurin (blue) to resorufin (violet) that can be detected via fluorescence measurement. For IC_{50} value determination, GraphPad Prism 7 (GraphPad Software Inc, San Diego, CA, USA) was used. Inhibitor curves were plotted with $Y = Bottom + (Top-Bottom)/(1+10^{((LogIC_{50}-X)*HillSlope)})$, with DMSO treatment as a positive control and medium only as a negative control. The background was subtracted from the measured values. Outliers were detected via the D'Agostino–Pearson omnibus normality test as mentioned in the kinase assay description. The assay was performed with three technical replicates.

2.6 Kamiokanne

The “super-kamiokande” (SKK) is used to investigate secondary cosmic radiation. The original SKK system consists of a huge water tank filled with 50,000 tons of ultra-pure water in which CR is generated by incoming neutrinos. The CR is detected by 13,000 photomultipliers that line the surface of the tank. In contrast, the simplified “kamiokanne” (KK) was originally developed at the University of Mainz and was then further established by the astroparticle project of the “Netzwerk Teilchenwelt” by the University of Göttingen and the “Deutsche Elektronen- Synchroton" DESY (Hamburg, Germany). The KK is composed of common thermos flasks with attached photomultiplier tubes (PMTs) and internal high-voltage supplies. Unlike the SKK, the KK measures not neutrinos but muons, which also move faster than light in an aqueous medium, thus generating CR as electromagnetic radiation, too. The PMTs are able to detect this CR, and consequently, an electric signal is generated as read-out. The actually mobile and relatively simple to handle KK is typically used in educational facilities to detect secondary cosmic irradiation. Obviously, if the KK can detect CR from muons, it should also be able to detect CR generated by radionuclides. Thus, we decided to test the KK for the detection of CR in our experiments. For this purpose, a volume of radioactive sample defined by the required activity was placed in a quartz cuvette and filled up with distilled water to the maximum volume of 3 mL. The tightly closed cuvette was placed in one of the thermos flasks completely filled with water. As a control, a second KK contained a cuvette filled with pure water for blank measurement to emit signals from the secondary cosmic rays. The cuvettes were made of quartz glass to make sure that the material was not absorbing the expected CR (250–600 nm wavelength range). We adjusted the sensitivity of signal detection so that signals from the CR in the control setup were just detectable (threshold emitted by muons from the secondary cosmic radiation). Then, we recorded the light signals entering the PMTs during measurement at 10-minute intervals. To verify whether the incoming signals in the experiment were actually based on the CR and not on the beta radiation itself, a thin layer of aluminum film (20 µm) was placed around the PMTs in a control experiment. In contrast to the beta irradiation, photons from CR were not able to penetrate the aluminum film. As a result, no signals were detected for this experiment, suggesting the read-out signal was not altered by the beta irradiation.

2.7 Radioactive experiments

2.7.1 *HPLC analysis*

Content determination of radioactive samples of ^{18}F, ^{18}FDG, and ^{68}Ga was performed using an Agilent 1100 HPLC with a DAD detector and column heating placed behind shielding (Agilent Technologies, Waldbronn, Germany). The column was a Chromolith® SemiPrep RP18 column (100 × 4.6 mm, Merck Millipore, Darmstadt, Germany). For analysis of samples with ^{90}Y, a Beckman (Beckmann Coulter, Krefeld, Germany) quaternary pump system gold 126, UV detector system Gold 166, and a Chromolith HR 4.6 × 100 mm column were used. The injection volume of all HPLC experiments was 5 µL. A gradient of acetonitrile and water, both with 0.1% (*v*/*v*) formic acid, was used as the mobile phase with a fixed flow rate (1.5 mL/min). UV detection at 296 nm was used for content determination.

2.7.2 *Beta Spectra of the chosen nuclides ^{18}F, ^{68}Ga, and ^{90}Y*

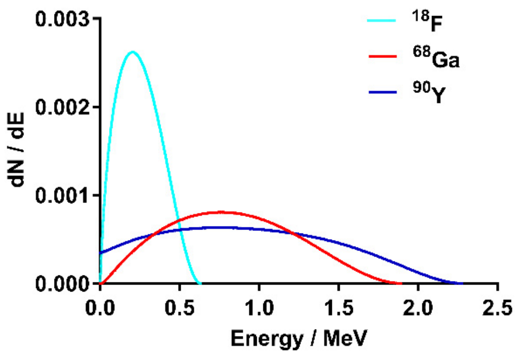

Supplemental Figure S8. Beta spectra of the nuclides ^{90}Y, ^{68}Ga, and ^{18}F used in this study. The emitting energies of the respective nuclides can be determined from the spectra. The energy levels that were mainly produced correspond to E_{mean} and are 0.25 MeV for ^{18}F, 0.84 MeV for ^{68}Ga, and 0.93 MeV for ^{90}Y. It is also possible to determine the maximum high energy level of ^{18}F with E_{max} = 0.63 MeV, ^{68}Ga with 1.90 MeV, and ^{90}Y with an E_{max} = 2.28 MeV. All data were obtained by Betashape (run with default parameters) using data from the International Atomic Energy Agency, Nuclear data services (https://www-nds.iaea.org/relnsd/vcharthtml/VChartHTML.html, accessed on July 6th 2021).

2.7.3 ^{18}F experiments

An aqueous solution of cagedAZD5438 (50 µM) was prepared in 10% DMSO. Briefly, 100 µL of the stock solution was transferred into a common amber glass HPLC vial (1.5 mL maximum volume). Furthermore, 900 µL of ^{18}F in water and 900 µL water for the negative control were added to the HPLC vial. Both samples were incubated in the autosampler of the HPLC at room temperature behind shielding.

2.7.4 ^{18}FDG experiments

Briefly, 200 µL from a 50 µM solution in 50% MeOH was transferred into a common amber glass HPLC vial (1.5 mL maximum volume). Furthermore, 200 µL of ^{18}FDG in water and 200 µL water for the negative control were added to the HPLC vial. Both samples were incubated in the autosampler of the HPLC at room temperature behind shielding.

2.7.5 ^{68}Ga experiments

A stock solution (250 µM) of cagedAZD5438 was prepared in DMSO. In brief, 250 µL of the stock solution was transferred into a common amber glass HPLC vial (1.5 mL maximum volume). Furthermore, 1000 µL of ^{68}Ga in 1 M HCl, prepared with a ^{68}Ga generator, and 1000 µL of 100 µM non-radioactive ^{69}Ga in 1 M HCl for the negative control were added to the HPLC vial behind shielding. The used radioactivity is indicated for each experiment. Both samples were incubated in the autosampler of the HPLC at room temperature behind shielding. Samples were injected to the HPLC alternatively and analyzed. Time points and peak areas are indicated for each experiment.

2.7.6 ^{90}Y experiments

To a 250 µM solution of cagedAZD5438 in DMSO, 420 MBq of ^{90}Y in 0.04 M HCL was added in a brown glass vial behind a lead wall. The solution was filled up to 1250 µL with water for injection. As no autosampler was available for this HPLC system, the solution was injected by hand in a 5 µL loop.

2.7.7 Stability tests of AZD5438 with the nuclides and under irradiation with 365 nm

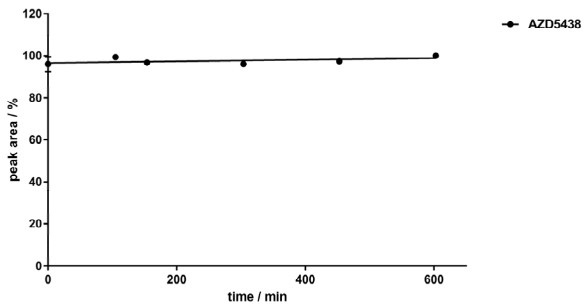

(**a**)

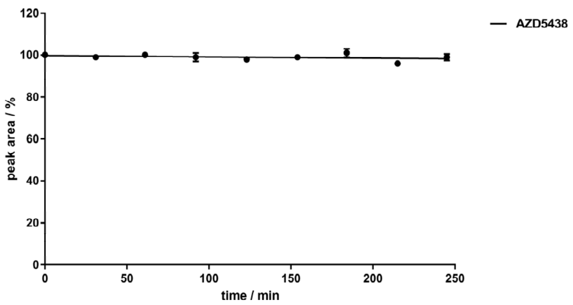

(**b**)

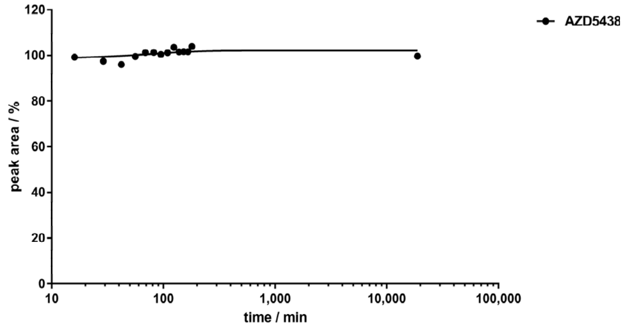

(**c**)

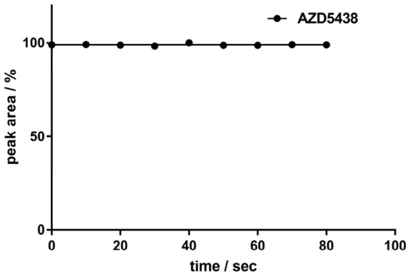

(d)

Supplementary Figure S9. Stability of AZD5438 in relation to the 3 isotopes (**a**) ^{18}Fluor, (**b**) ^{68}Gallium, and (**c**) ^{90}Yttrium and under UV light of (**d**) 365 nm, 37.5 mW, every 10 seconds. Each value is the mean ± SD of three independent experiments.

2.8 Linear particle accelerator experiments

Linear particle accelerators are established for external beam radiotherapy in clinical applications. Irradiation caused by a linear particle accelerator might be capable of generating CR due to the high-energy charged particles such as electrons or photons traveling through aqueous media. Particle beams from linear particle accelerators are able to reach deep tissues with a very precise local resolution. In 2011, Axelsson et al. demonstrated through optical imaging that radiation from a clinical linear accelerator produced CR in aqueous tissue phantoms of both electrons and photons, which increased with higher radiation energy (6–18 MeV). Therefore, in the project, we investigated whether irradiation from a linear particle accelerator was able to uncage cagedAZD5438 analogously to the concept using the radioisotopes. However, it was not possible to apply the KK for the direct detection of CR in the linear particle accelerator settings. Therefore, in a preliminary experiment, we exposed 50 μM cagedAZD5438 dissolved in 1 mL methanol in a translucent HPLC vial to a 6 MeV beam of photons with a maximum energy dose of 10 Gy, applied for 3-5 min. As positive controls, we irradiated samples with UV light following the irradiation from the linear particle accelerator. A cagedAZD5438 sample without irradiation was analyzed as a negative control. The subsequent HPLC analysis determined that the irradiation of the 6 MeV beam of photons caused no uncaging of cagedAZD5438 (Table S2, Figure S10). Next, we performed a second experiment following the previous settings but employing high-energy electron radiation. Here, we placed a 50 μM solution of cagedAZD5438 in 1.5 mL water with 25% DMSO into a 6 MeV electron beam of a linear particle accelerator, and a maximum radiation dose of 3 Gy was applied. Again, the subsequent HPLC analysis proved no uncaging of cagedAZD5438 under these conditions. In addition, uncaging did not occur by irradiating a respective 4-milliliter solution of cagedAZD5438 by electrons with a significantly higher energy of 18 MeV. Taken together, our results from these experiments utilizing photons and electrons from a linear particle accelerator suggest that not enough CR is induced under the chosen conditions and is thus not sufficient to cleave the PPG from the prodrug cagedAZD5438.

Supplementary Table S2. Irradiation of solutions of cagedAZD5438 (samples 1-3) using external particle beams from a linear particle accelerator. Three different experiments, each performed in duplicate, showed no uncaging of cagedAZD5438 in contrast to the positive controls irradiated with UV light of 365 nm.

Sample	Radiation	Energy /MeV	Maximum dose /Gy	Solvent	Total volume /mL	Compound concentration /μM
**1**	Photons	6	10	MeOH	1.0	50
**2**	Electrons	6	3	25% DMSO in water	1.5	50
**3**	Electrons	18	3	25% DMSO in water	4.0	50

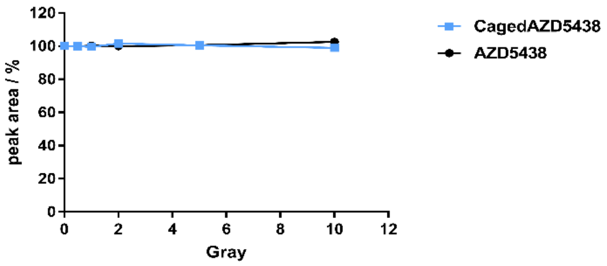

(**a**)

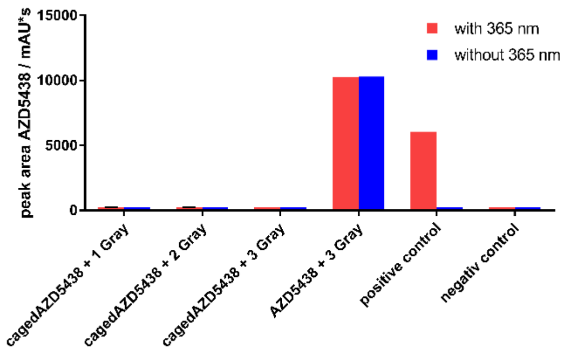

(**b**)

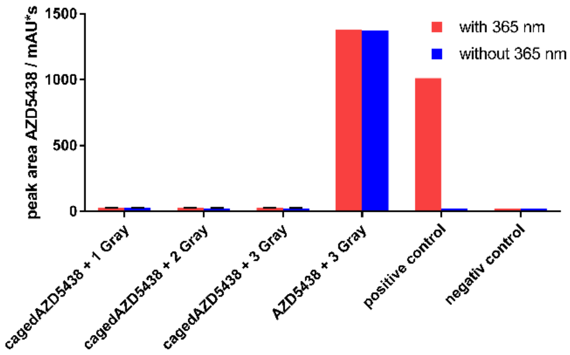

(**c**)

Supplementary Figure S10. Irradiation of a 50 µM solution of cagedAZD5438 by a linear particle accelerator under varying conditions. (**a**) Irradiation with 6 MeV photons (sample 1); (**b**) irradiation with 6 MeV electrons (sample 2); (**c**) irradiation with 18 MeV electrons (sample 3). As positive controls, following the irradiation with the line particle accelerator, samples 1-3 were irradiated with UV light of 365 nm in wavelength and thus photoactivated, showing uncaging to yield AZD5438. A sample of a solution of cagedAZD5438 as a negative control was kept in the dark.

2.8.1 Implementation

Activation experiments using a linear particle accelerator were performed using the electron linear accelerator Artiste (Siemens Medical Systems, Munich, Germany). Compound solutions (50 µM) were prepared according to Table S2 in either a clear glass HPLC vial (Experiments 1 and 2) or in a 10-milliliter clear glass vessel (Experiment 3). The vessels or HPLC vials were placed without a cap in the particle beam of the linear particle accelerator and irradiated with the respective radiation, energy, and maximum doses. The peak areas of both the caged and uncaged species were determined by HPLC analysis before and after irradiation. Additionally, both negative and positive samples were measured. The positive samples were irradiated with UV light by an LED (5.4 W, 365 nm, 2 min) after irradiation using the linear particle accelerator.

2.9 ESR experiments

The test compounds AZD5438 and cagedAZD5438 dissolved in DMSO (50 µM) were mixed 1:1 with the spin probe 2,3,7,8-Tetramethoxythianthrene (TMTH) in water (2.5 mM). The water contained EDTA (1 mM) to complex metal ions to avoid interference with ROS. As a positive control, the photosensitizer 1,4-naphthoquinone (MB) in DMSO was mixed with the spin probe TMTH to obtain a concentration of 50 µM and 2.5 mM of the latter. As negative controls, the substances were measured either without the use of the spin probe or without prior irradiation. The samples were irradiated with a 16 LED lamp for a duration of 100 s and at an intensity of 37.5 mW/well.

Spin probing experiments were carried out using an ELEXSYS E 500 system (Bruker Biospin, Rheinstetten, Germany). For acquisition of the ESR spectra, samples were taken from the 96-well plate using 25 µL ringcaps® (Hirschmann, Eberstadt, Germany). The ringcap® was then placed in an ESR tube and put into the cavity of the spectrometer. The measurements were taken in duplicate of two distinct samples using the X-band and the following parameters: center field, 3511.35 GHz; resonance frequency, 9.85 GHz; microwave power, 20 mW; modulation amplitude, 1.0 G; modulation frequency, 100 kHz; conversion time, 40 ms; time constant, 40.96 ms.
